# Localized surface plasmons in structures with linear Au nanoantennas on a SiO_2_/Si surface

**DOI:** 10.3762/bjnano.7.145

**Published:** 2016-10-26

**Authors:** Ilya A Milekhin, Sergei A Kuznetsov, Ekaterina E Rodyakina, Alexander G Milekhin, Alexander V Latyshev, Dietrich R T Zahn

**Affiliations:** 1 Novosibirsk State University, Pirogov 2, 630090, Novosibirsk, Russia; 2 Rzhanov Institute of Semiconductor Physics RAS, Lavrentiev Ave. 13, 630090, Novosibirsk, Russia; 3 Rzhanov Institute of Semiconductor Physics RAS, Novosibirsk Branch “TDIAM”, Lavrentiev Ave. 2/1, Novosibirsk, 630090, Russia; 4 Semiconductor Physics, Technische Universität Chemnitz, Chemnitz, Germany

**Keywords:** nanoantenna array, localised surface plasmon resonance, plasmon–phonon interaction, phonons, SiO_2_

## Abstract

The study of infrared absorption by linear gold nanoantennas fabricated on a Si surface with underlying SiO_2_ layers of various thicknesses allowed the penetration depth of localized surface plasmons into SiO_2_ to be determined. The value of the penetration depth derived experimentally (20 ± 10 nm) corresponds to that obtained from electromagnetic simulations (12.9–30.0 nm). Coupling between plasmonic excitations of gold nanoantennas and optical phonons in SiO_2_ leads to the appearance of new plasmon–phonon modes observed in the infrared transmission spectra the frequencies of which are well predicted by the simulations.

## Introduction

Plasmonic metamaterials remain the object of keen interest both in fundamental and applied research due to their unique optical properties including negative and zero refraction, focusing, filtering, polarization manipulation, etc. [1–4]. They are considered as perspective solutions for possible device applications that involve super- and hyperlenses, and for energy concentrators, cloaking materials, sensors, and others. The morphology of the metastructures can be varied from simple planar elements to more complicated three-dimensional structures. Linear nanoantennas are commonly used in optical sensors due to relative simplicity of their fabrication [5–9]. At the same time, as compared to alternative nanoantenna geometries, the linear nanoantennas are highly demanded in sensing as they provide maximal local field amplification which is of prime importance for enhancing the optical response of the structure.

In a conventional design, the linear nanoantenna structures are represented by a 2D array of periodically arranged rods of metal (e.g., Au, Al, Ag) the typical length of which falls into the range from tens of nanometers to a few micrometers, while having a width of about 100 nm that is defined by conventional nanolithography used for nanoantenna fabrication. Such nanoantennas exhibit the effect of the localized surface plasmon resonance (LSPR), which is observed when the eigenfrequency of electron oscillations in nanoantenna coincide with the frequency of the exciting electromagnetic radiation. The LSPR yields a sharp increase of the local electromagnetic field magnitude near the nanoantenna surface that makes feasible to detect a small amount of alien substances located in the near field region of the nanoantenna [8–10].

The linear nanoantennas have uniaxial symmetry and, therefore, can be characterized by two LSPR modes. The transverse mode is polarized perpendicular to the antenna and has the energy localized in the optical domain of the electromagnetic spectrum. The energy of the longitudinal mode, which is polarized along the antenna, depends on the structural parameters (the length, width, and height of the antennas, as well as the distance between them), the dielectric function of surrounding media and substrate materials, and can be varied within a wide spectral range from visible to far infrared or terahertz frequencies [11–14]. Nanoantennas exhibiting the LSPR in the optical spectral range are already used for surface-enhanced Raman scattering (SERS) [15–19], and for fluorescence enhancements [20–22]. Nanoantennas with the LSPR energy located in the infrared spectral region are considered as promising nanostructures for the detection of small amounts of both organic (down to attomoles) and inorganic substances, including semiconductor nanocrystals [18,23–27]. In this approach serving as the basis for surface-enhanced IR spectroscopy, the IR response of various thin films and adsorbents due to coincidence of LSPR energies in the nanoantenna and vibrational states of a studied substance [28–30].

When the LSPR energy in metal nanostructures is close to the optical phonon energy of investigated substances or thin films, the effects of plasmon–phonon coupling are to be expected. Indeed, as it was is shown in [31], such a coupling may drastically increase (by a factor of 200) the scattering intensity in the near field of the metal (Pt) tip of an atomic force microscope at frequencies of the SiC surface optical (SO) phonons. The results of the theoretical study of coupling between the LSPR and SO phonon modes in the nanoantenna structures formed on a GaN substrate were also reported [32]. A significant increase of the phonon response (1900 times) at the SO phonon frequencies is observed for the structures with nanoantenna arrays fabricated on a 3 nm thick natural silicon oxide layer. This phenomenon is interpreted in terms of the coupling between the antenna's, plasmon resonance and surface phonon–polariton excitations [26]. Similar results were obtained for a thermal SiO_2_ layer having the thickness of 106 nm, for which the SO phonon mode at 1230 cm^−1^ was investigated [10]. Very recently, G. Cacciato et al. [33] have observed plasmon–phonon modes in TiO_2_ films with embedded Ag nanoparticles due to the coupling of longitudinal optical (LO) phonons in TiO_2_ with free carriers present in the vicinity of Ag nanoparticles.

It should be noticed that available literature exhibits a lack of information on the dependence of the LSPR energy on the oxide layer thickness and the LSPR localization depth. This paper presents a systematic experimental and numerical study for the dependence of the LSPR energy on the structural parameters of the gold nanoantenna arrays formed on Si substrates with SiO_2_ sublayers of a variable thickness. The character of this dependence allows establishing the peculiar features of plasmon-phonon interaction in the structures and determining the LSPR depth localization.

## Experimental

The linear nanoantenna arrays with the aforementioned structural parameters providing the longitudinal LSPR energy in the range of 600–4500 cm^−1^ were fabricated using electron beam nanolithography. We employed the directly writing nanolithographic machine Raith-150 (Raith GmbH, Germany) using technological steps described in [34]. Because of technological restrictions, the operating area of the fabricated nanoantenna arrays was limited by 3 × 3 mm^2^. The structural parameters of the nanoantennas were controlled by scanning electron microscopy.

The IR transmission spectra of the fabricated Au nanoantenna arrays were measured in the frequency range of 600–4500 cm^−1^ by means of the FTIR spectrometer Bruker IFS-113v. The spectrometer provided the spectral resolution of 4 cm^−1^ and was combined with an IR microscope enabling to focus the infrared radiation to a spot with a diameter of 100 µm. A globar (SiC) was utilized as a source of IR radiation, which was registered with a cooled HgCdTe detector. The IR spectra of the nanoantenna structures were normalized to the signal from the substrate without nanoantennas. The measurements were carried out at room temperature.

The thickness of the SiO_2_ layers was determined by a conventional ellipsometric technique which provided an accuracy of 1 nm in the thickness evaluation.

## Results and Discussion

**Figure 1:**
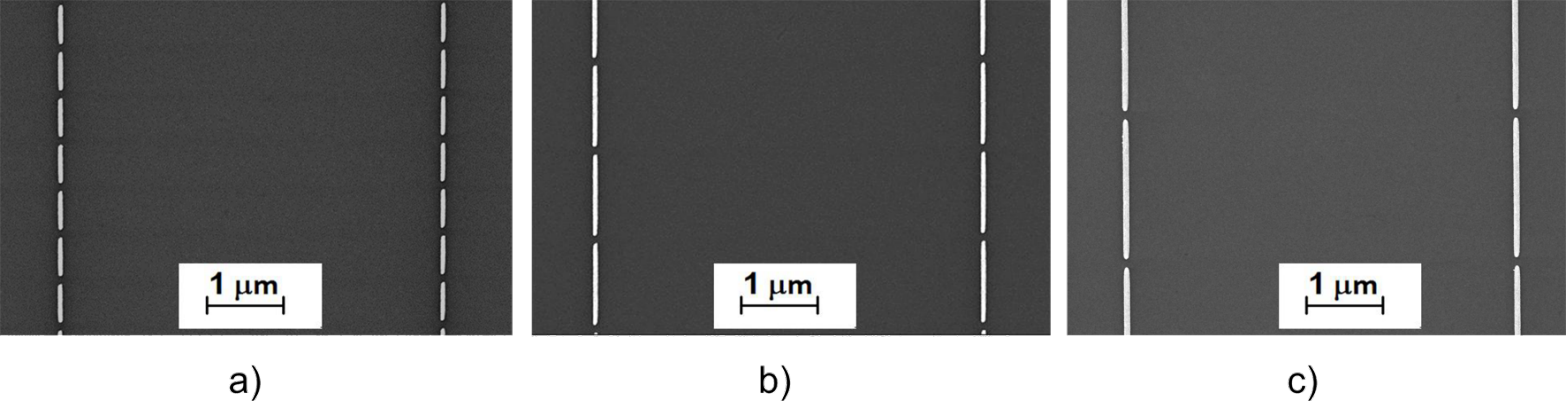
Typical SEM images of the fabricated Au nanoantennas for the mid-IR spectral region. Nanoantenna lengths: a) 500 nm, b) 1100 nm, c) 1900 nm.

In this work, a set of uniform nanoantenna arrays with different lengths ranging from 500 to 1900 nm was fabricated by using nanolithography. Typical SEM images of the resulting structures are illustrated in Figure 1. The nanoantennas had a height of 50 nm, which was specified by the thickness of the gold layer deposited in the nanolithographic process.

A relatively large transverse period of nanoantennas (ca. 4 μm) was chosen to exclude the interaction between neighboring Au nanoantennas in the transverse direction. On the contrary, in the vertical direction the gap between nanoantennas was as small as 100 nm to maximize their mutual coupling and intensify the electric field inside the gap. The same value of 100 nm was imposed on the nanoantenna width.

**Figure 2:**
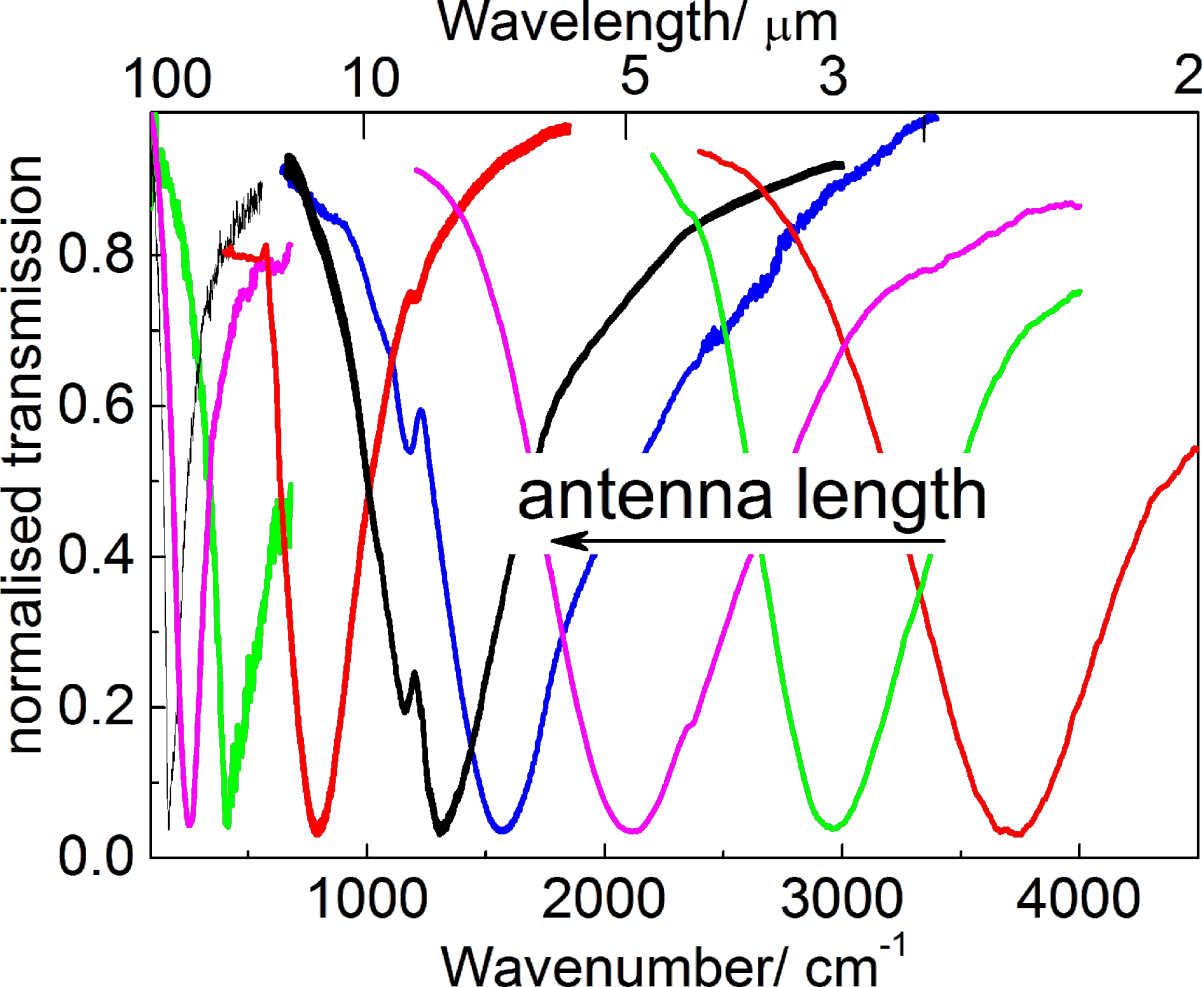
Typical IR transmission spectra of linear antennas with different lengths.

IR transmission spectra of the fabricated nanoantennas described above are shown in Figure 2. The spectra demonstrate distinct deep minima, the position of which corresponds to the LSPR energy.

**Figure 3:**
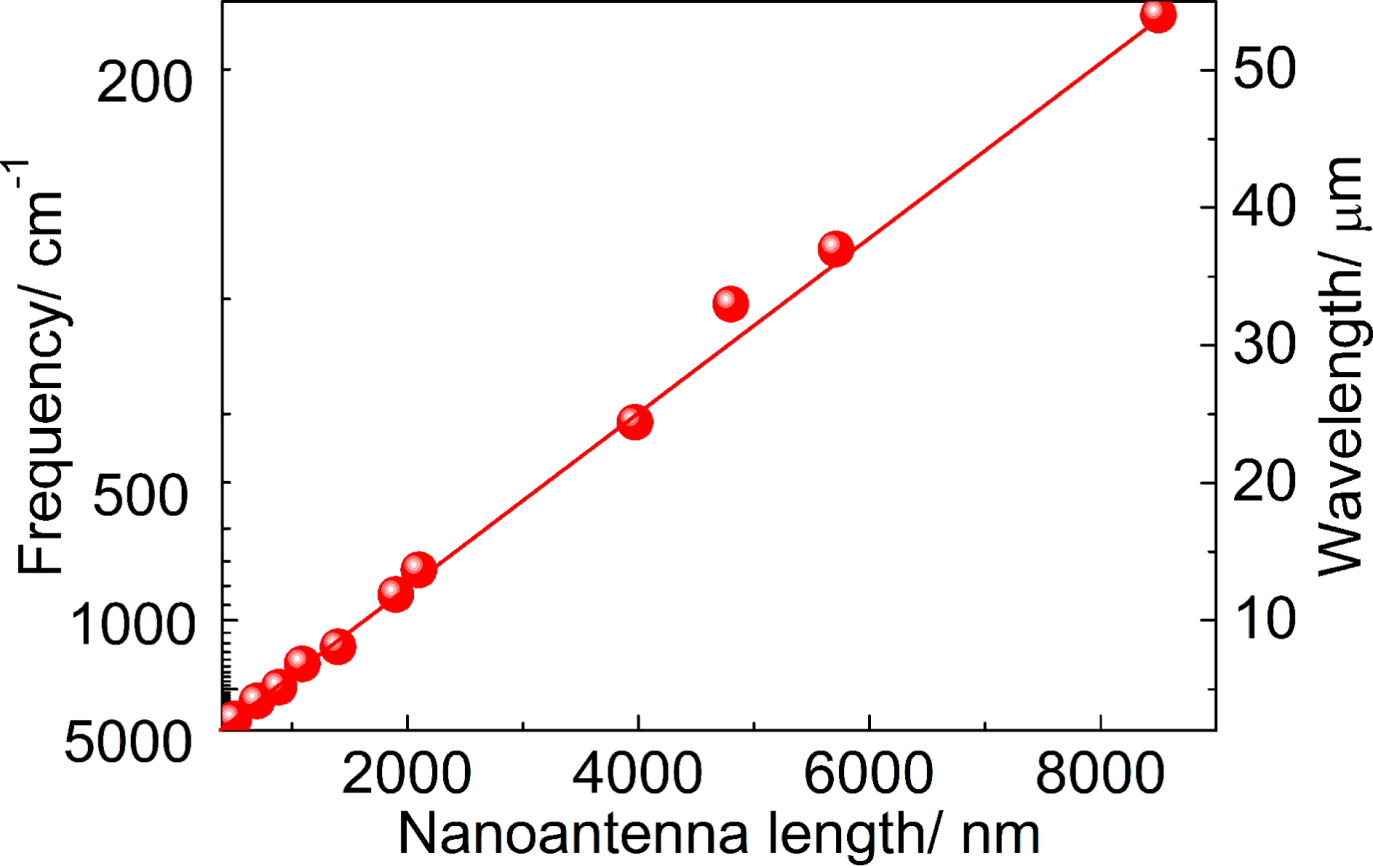
LSPR energy in nanoantenna arrays fabricated on bare Si surfaces as a function of the nanoantenna length.

As predicted earlier [35], the experimentally determined LSPR wavelength depends linearly on the antenna length in a wide spectral range (Figure 3). Such behavior originates from the dipole resonance phenomenon, which states that linear nanoantennas effectively couple to the incident electromagnetic waves polarized along the nanoantenna axis when their wavelength coincides with the doubled antenna length [36]. This coupling also depends on the dielectric function of a surrounding medium that makes the LSPR energy different for nanoantennas backed by a bare Si substrate and when a SiO_2_ sublayer is introduced beneath the nanoantennas.

**Figure 4:**
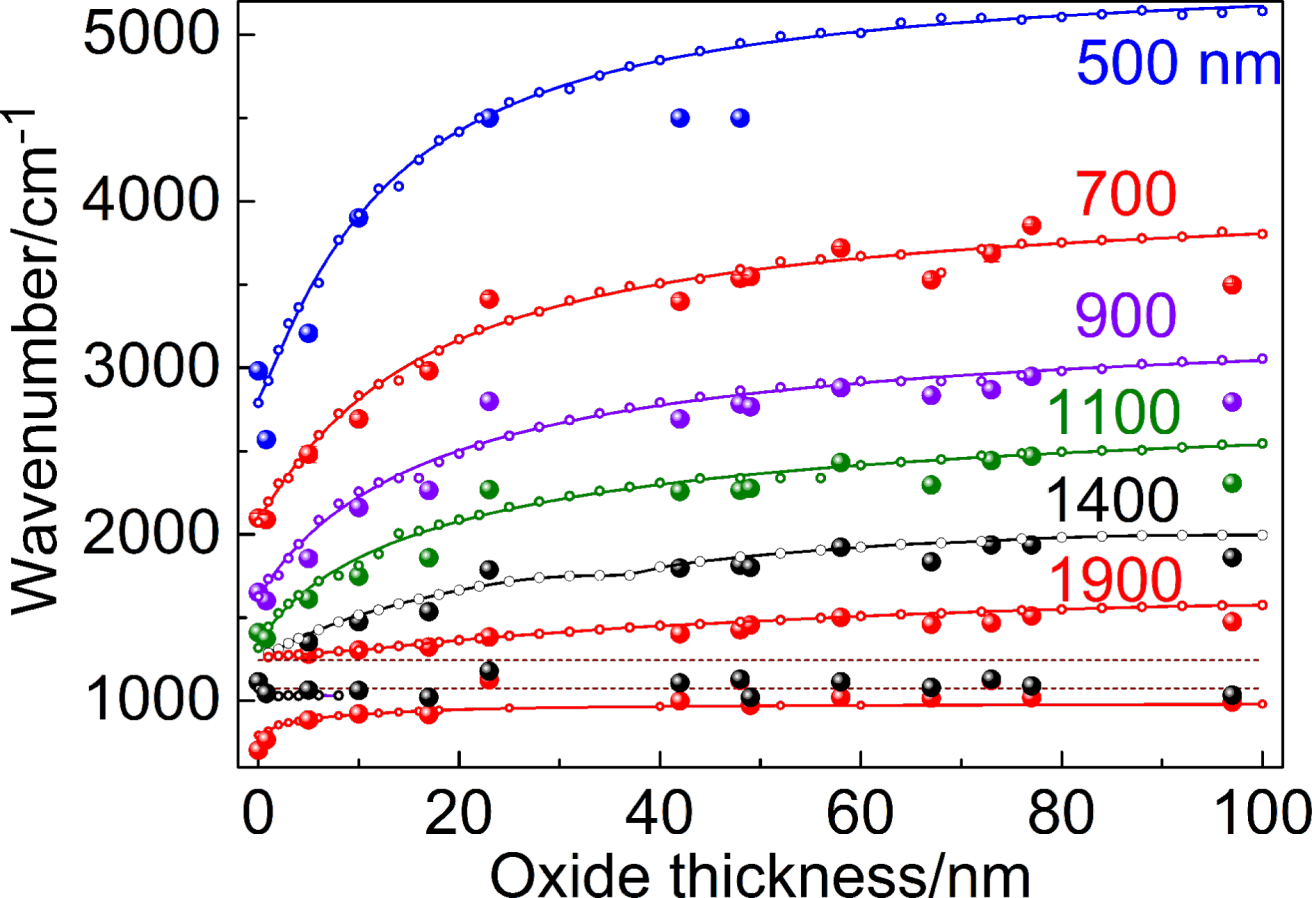
LSPR frequencies of nanoantenna arrays versus the SiO_2_ thickness. Six structures with different antenna lengths ranging from 500 to 1900 nm are presented. Large circles: experiment; solid lines with dots: full-wave simulations.

Figure 4 illustrates the relation between the LSPR frequency and the SiO_2_ layer thickness derived from the IR spectra of nanoantennas with the underlying SiO_2_ layer created on top of the Si substrate. It can be seen from Figure 4 that the LSPR frequency of the nanoantennas fabricated on a thick SiO_2_ layer is blue-shifted by about 1000 cm^−1^ with respect to the structures on bare Si due to the significant difference between the dielectric functions of Si and SiO_2_. With decreasing SiO_2_ layer thickness, starting from about 30 nm, a rapid transition from the LSPR energies derived for nanoantennas fabricated on a thick SiO_2_ layer, to the corresponding value in antennas on bare Si is observed (Figure 4). This is due to the fact that the effective dielectric function of the surrounding medium for the borderline case should be considered as a combination of the weighted dielectric functions of Si and SiO_2_ [37]. Thus, the oxide thickness of 20 ± 10 nm, for which significant changes in the LSPR frequency occur, defines the LSP penetration length.

In order to accurately examine influence of the SiO_2_ sublayer on the LSPR properties, the 3D full-wave electromagnetic simulations using ANSYS HFSS ™ v.15 software [38] were carried out in this work and distributions of the electromagnetic field near the nanoantenna surface were numerically studied. To model the nanoantenna array as a uniform periodic structure, we exploited a regime of Floquet ports and periodic boundary conditions applied to the structure unit cell. The nanoantennas were considered to be supported by a thin SiO_2_ layer with a thickness less than or equal to 100 nm formed on a silicon substrate. The dielectric functions of SiO_2_ and Si used in the simulations were taken from [37]. Gold was modeled as a lossy dispersive medium with the dielectric permittivity ε_Au_ described by the classical Drude formula:


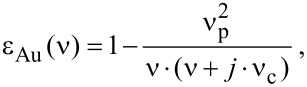


where ν is the radiation frequency, and ν_p_ ≈ 72500 cm^−1^ and ν_c_ ≈ 216 cm^−1^ are the linear plasma frequency and the damping frequency, respectively [39].

**Figure 5:**
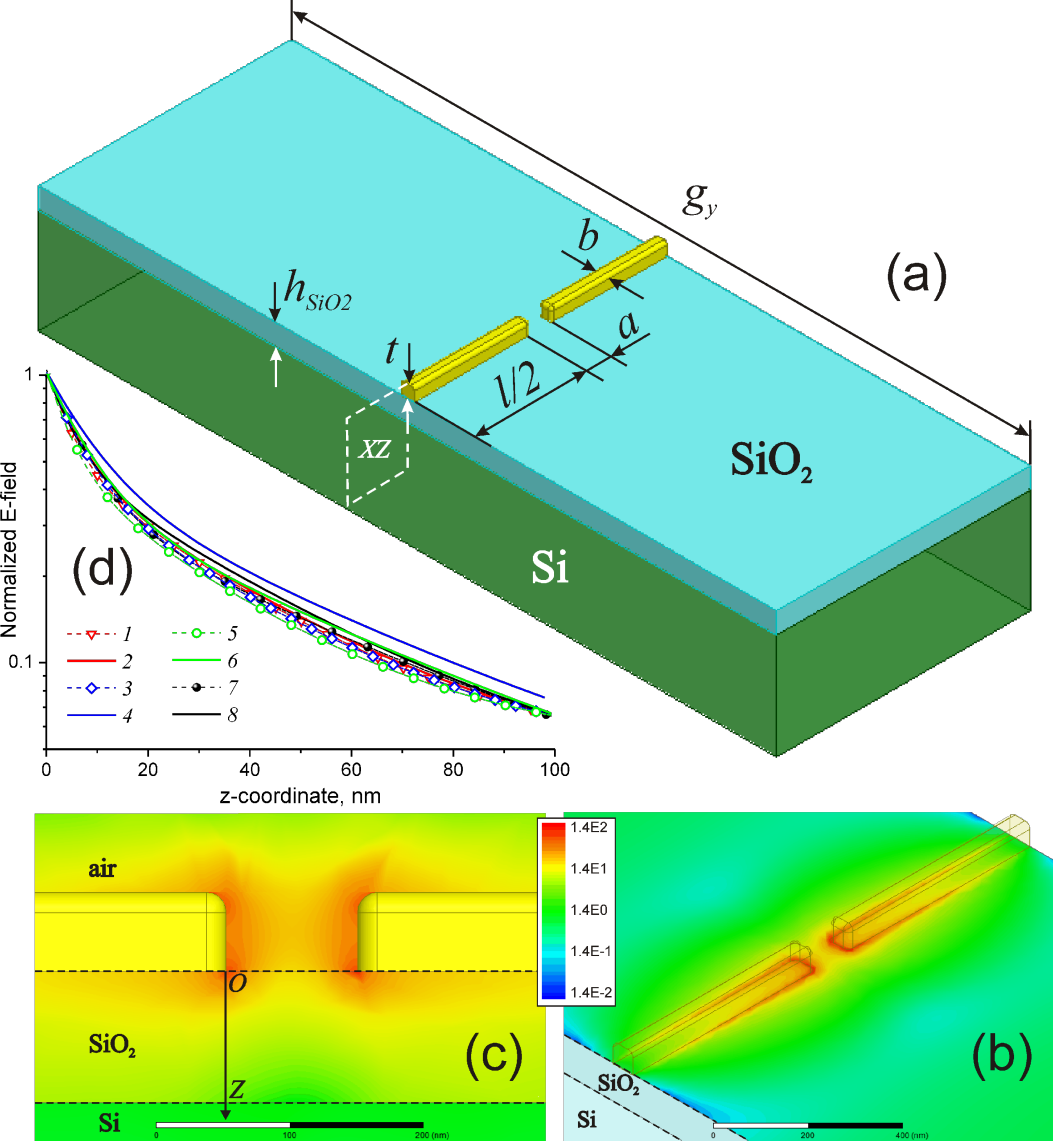
a) Structure geometry definition for the nanoantenna array (the unit cell is shown). b) Example of the distribution for the normalized electric field magnitude over SiO_2_ surface for the nanoantenna length *l* = 1100 nm. c) Similar *E*-field distribution referred to the vertical plane (XY) cutting the nanoantenna in the middle. d) Functional behavior of the near field decay along the *OZ* axis at the longitudinal LSPR frequency calculated for different nanoantenna lengths: 1: *l* = 500 nm, *h *_SiO2_ = 0 nm; 2: *l* = 500 nm, *h*_SiO2_ = 100 nm; 3: *l* = 700 nm, *h*_SiO2_ = 0 nm; 4: *l* = 700 nm, *h*_SiO2_ = 100 nm; 5: *l* = 900 nm, *h*_SiO2_ = 0 nm; 6: *l* = 900 nm, *h*_SiO2_ = 100 nm; 7: *l* = 1100 nm, *h*_SiO2_ = 0 nm; 8: *l* = 1100 nm, *h*_SiO2_ = 100 nm.

In the simulations, the width *b* and the height *t* of the nanoantennas were assumed to be 50 nm with an the axial gap of *a* = 100 nm (see Figure 5a) as it was imposed by our nanofabrication. The nanoantennas were assigned to have rounded edges with a fillet radius of 15 nm. The thickness *h*_SiO2_ of the SiO_2_ layer was a variable parameter ranging within 0–100 nm. The transverse pitch *g_y_* of the nanoantennas was fixed as 5000 nm, while the nanoantenna lengths *l* of 500, 700, 900, 1100, 1400, and 1900 nm chosen for practical implementation were used in numerical simulations. The listed nanoantenna lengths (1400 and 1900 nm) secure the proximity of ther LSPR energy to the energy of optical phonons in SiO_2_. For the fixed structural parameters of the nanoantenna array, the simulations were aimed at determining the longitudinal LSPR frequency from the calculated IR transmission spectra of the structures with nanoantennas as a function of the SiO_2_ thickness. The results of these simulations are shown in Figure 4 (solid lines with dots). Quite good agreement between simulations and experiment can be deduced from the figure.

Figure 5b illustrates the distribution of the electric field magnitude over the SiO_2_ surface within a unit cell of the nanoantenna array by the example of the antenna length *l* = 1100 nm and the SiO_2_ thickness *h*_SiO2_ = 100 nm. The distribution is simulated for the frequency of the longitudinal LSPR (2558 cm^−1^) and implies that the nanoantennas are excited normally by the electromagnetic wave polarized linearly along their axis. The field magnitude is normalized to that when the nanoantennas are removed from SiO_2_ surface, thereby yielding the relative *E*-field amplification distribution. Similarly, Figure 5c represents the simulated *E*-field distribution referred to the middle vertical plane (XY) and shows that the field penetrates to the SiO_2_ layer at a relatively small distance. Quantitatively, the LSPR penetration depth δ_LSPR_ in Si and SiO_2_ can be derived from Figure 5d, which shows plots of the *E*-field decay along the *OZ* axis simulated for different antenna lengths by the example of the two marginal cases: *h*_SiO2_ = 0 and 100 nm. As retrieved from the plots, the typical value of the *E*-field decay by the factor of *e*, herein referred to as δ_LSPR_, falls within 12.5–13.9 nm and 14.4–16.6 nm for *h*_SiO2_ = 0 and 100 nm, respectively. It is noteworthy that functionally the *E*-decay behavior is fitted well not by a single exponent but by a sum of two exponents *P*_1_•exp(*−z*/δ_1_) and *P*_2_•exp(*−z*/δ_2_), which describe short-range (near-field) and long-range (far-field) components of the LSPR field, respectively [5]. For the presented plots, the values of δ_1_ and δ_2_ are correspondingly ranged within 4.7–8.9 nm and 30.1–51.3 nm, while the exponent amplitudes are characterized by the ratio *P*_1_/*P*_2_ ≈ 1.15–2.00 indicating predominance of the short-range field component.

When the nanoantenna structure is located on the interface between two semi-infinite dielectric media with the dielectric permittivities ε_1_ and ε_2_, its LSPR frequency ν_ε1,ε2_ undergoes a red shift relative to the similar frequency ν_1,1_ when nanoantennas are placed in free space. These frequencies are linked via the effective refractive index *n*_eff_ = [(ε_1_ + ε_2_)/2]^1/2^: ν_ε1,ε2_ = ν_1,1_/*n*_eff_ [12]. This allows one to conclude that when the nanoantennas are backed by a SiO_2_ layer the thickness of which is noticeably larger than the LSPR penetration depth δ_LSPR_, the following relation between the LSPR frequencies ν_Si_ and ν_SiO2_ corresponding to the cases of bare Si (*h*_SiO2_ = 0) and thick SiO_2_ layers should be valid:

[1]
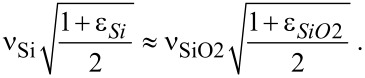


Using data from Figure 4, it can be verified that for *h*_SiO2_ = 100 nm Equation 1 is satisfied with an accuracy of 5–7%.

It is noteworthy that the experimental and numerical data from Figure 4 are fitted well by allometric functions (solid lines) of the following kind:


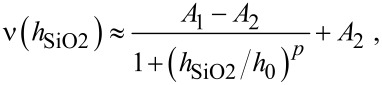


where *A*_1_, *A*_2_, *p*, *h*_0_ are the constants. For the investigated nanoantenna structures, excluding the case of *l* = 1900 nm complicated by plasmon–phonon interactions, the exponent *p* changes from 1.15 down to 0.7 when the length varies from 500 to 1400 nm, while the characteristic length *h*_0_ of the allometric function alteration changes within 12.9–30.0 nm. The latter values are assessed to be in good concordance with the LSPR localization depth δ_LSPR_ and the “two exponents fit” established by us in numerical simulations.

**Figure 6:**
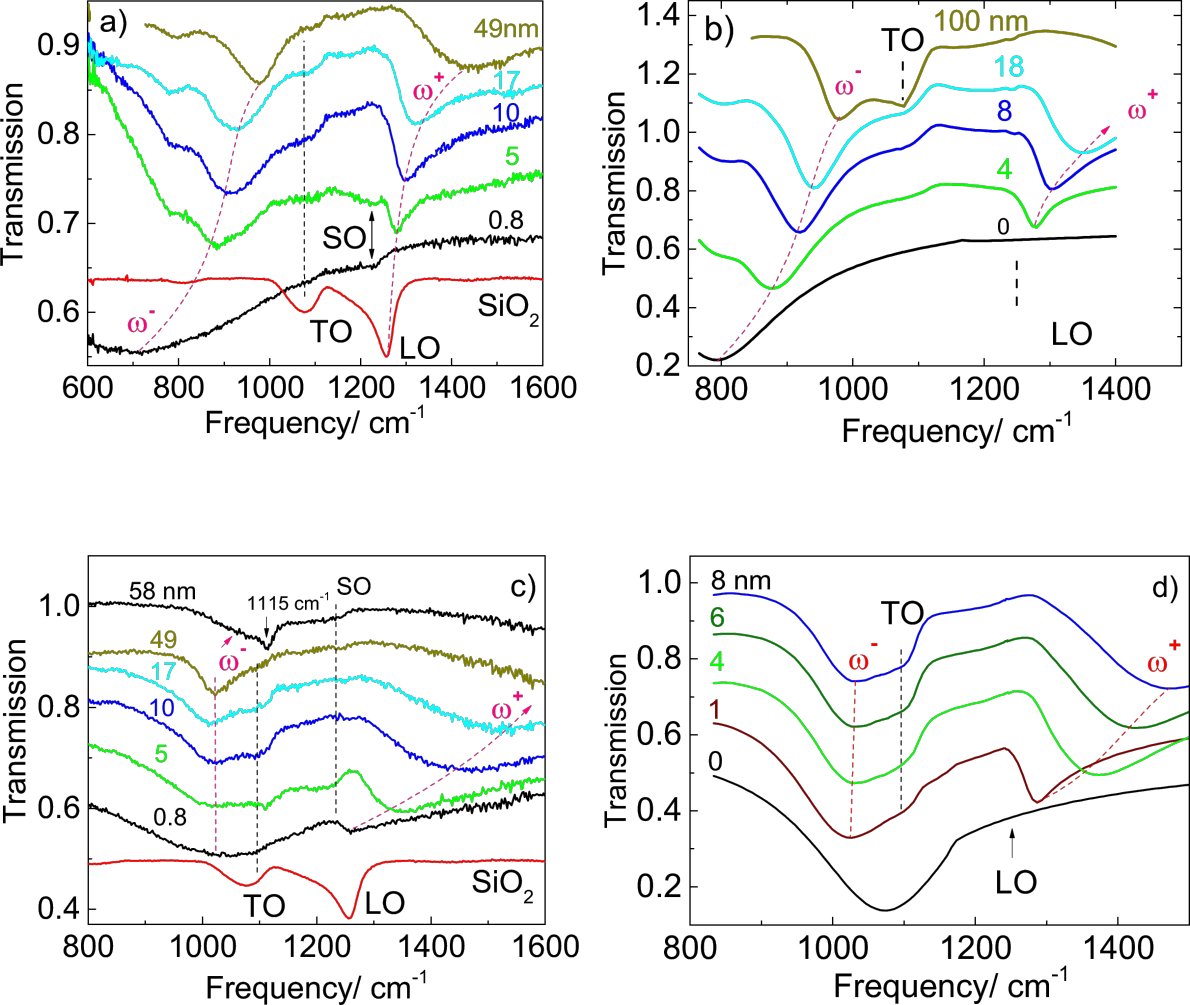
Experimental (a,c) and calculated (b,d) IR transmission spectra of nanoantenna array with the lengths of 1900 nm (a,b) and 1400 nm (c,d) fabricated on SiO_2_ layers of different thicknesses measured at normal incidence. The IR transmission spectrum of a 49 nm thick SiO_2_ layer on a Si substrate measured at off-normal (70°) incidence is shown for comparison. The vertical dashed lines indicate the frequency position of the TO, SO, and LO phonons in SiO_2_.

It is important to highlight that the influence of the SiO_2_ layer thickness on the LSPR frequency of nanoantennas with the length of 1400 and 1900 nm becomes more complex in the vicinity of the TO and LO phonon frequencies in SiO_2_, which are 1070 and 1240 cm^−1^, respectively. The experimental and calculated IR transmission spectra of these structures are presented in Figure 6. As one can see from the figure, the IR spectrum of the structure with the thickness of a natural silicon oxide layer of 0.8 nm reveals a pronounced minimum at 700 cm^−1^ corresponding to the LSPR mode. A weaker feature located between the TO (1075 cm^−1^) and LO (1250 cm^−1^) phonon modes in SiO_2_ at 1230 cm^−1^ is assigned as a SO phonon mode according to earlier observations for nanoantennas on thin SiO_2_ [25]. However, with the increasing SiO_2_ layer thickness the position of LSPR is shifted towards higher frequencies. At a thickness of about 5 nm the LSPR mode splits into two modes. The low frequency mode (ω^−^) located at frequencies below the TO phonon modes approaches the frequency position of the TO phonon with increasing SiO_2_ layer thickness. The high frequency mode (ω^+^) splits off from the LO phonon frequency reaching the value of 1280 cm^−1^ for 5 nm thick SiO_2_. With further increase of the SiO_2_ layer thickness the ω^+^ mode asymptotically approaches the LSPR mode frequency. The calculated IR spectra shown in Figure 6b,d describe well the experimental results.

**Figure 7:**
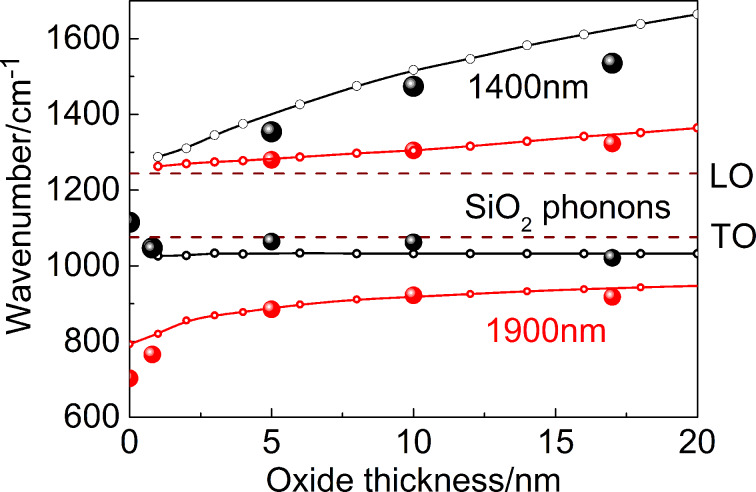
Splitting the LSPR modes due to plasmon–phonon interaction for nanoantennas with the lengths of 1400 and 1900 nm. Large circles: experiment; solid lines with dots: full-wave simulations.

The splitting of the LSPR modes due to the plasmon–phonon interaction is additionally illustrated in Figure 7, wherein the experimental points (circles) and simulated curves (solid lines with dots) show the frequency positions for the ω^−^ and ω^+^ modes as a function of the SiO_2_ sublayer thickness plotted in the spectral range of optical phonons in SiO_2_. The graphs correspond to the nanoantenna length of 1400 and 1900 nm and reproduce Figure 4 at a larger scale. Note, the spectral splitting is a characteristic feature of coupling between plasmon excitations and optical phonons in heavily doped ionic semiconductors [40–41]. Here, instead of 3D plasmon excitations of free electron gas in doped semiconductors, we deal with the localized surface plasmon excitations induced by Au nanoantennas that interact with the bulk optical phonons in the SiO_2_ layer. Moreover, the SiO_2_ layer thickness governs the LSPR mode frequency in a similar manner as the free-carrier concentration specifies the plasmon frequency in ionic materials.

## Conclusion

In this work, the dependence of the LSPR frequency on the thickness of the underlying SiO_2_ layer are thoroughly studied via analyzing the experimental and simulated IR transmission spectra for the arrays of linear nanoantennas with different lengths. The character of this dependence allowed us to determine the value of the penetration depth of about 20 nm for the localized surface plasmon in the SiO_2_ layer. It is found that the plasmon–phonon interaction leads to splitting the LSPR mode into two branches (low and high frequency) when its energy approaches to those of optical phonons in SiO_2_. The experimental data are demonstrated to be in good concordance with full-wave simulations in ANSYS HFSS™ electromagnetic software.

## References

[1] Cai W, Shalaev V (2010). Optical Metamaterials: Fundamentals and Applications.

[2] Pendry J B (2000). Phys Rev Lett.

[3] Belov P A, Simovski C R, Ikonen P (2005). Phys Rev B.

[4] Porterfield D W, Hesler J L, Densing R, Mueller E R, Crowe T W, Weikle R M (1994). Appl Opt.

[5] Adato R, Altug H (2013). Nat Commun.

[6] Alonso-González P, Albella P, Neubrech F, Huck C, Chen J, Golmar F, Casanova F, Hueso L E, Pucci A, Aizpurua J (2013). Phys Rev Lett.

[7] Selig O, Siffels R, Rezus Y L A (2015). Phys Rev Lett.

[8] Pucci A, Neubrech F, Aizpurua J, Cornelius T, Lamy de la Chapelle M, Wang Z (2008). Electromagnetic Nanowire Resonances for Field-Enhanced Spectroscopy. One-Dimensional Nanostructures.

[9] Lamy de la Chapelle M, Pucci A (2013). Nanoantenna: Plasmon - Enhanced Spectroscopies for Biotechnological Applications.

[10] Levin C S, Kundu J, Barhoumi A, Halas N J (2009). Analyst.

[11] Biagioni P, Huang J-S, Hecht B (2012). Rep Prog Phys.

[12] Neubrech F, Kolb T, Lovrincic R, Fahsold G, Pucci A, Aizpurua J, Cornelius T W, Toimil-Molares M E, Neumann R, Karim S (2006). Appl Phys Lett.

[13] Ayas S, Topal A E, Cupallari A, Güner H, Bakan G, Dana A (2014). ACS Photonics.

[14] Razzari L, Toma A, Shalaby M, Clerici M, Zaccaria R P, Liberale C, Marras S, Al-Naib I A I, Das G, De Angelis F (2011). Opt Express.

[15] Nie S, Emory S R (1997). Science.

[16] Grand J, Lamy de la Chapelle M, Bijeon J-L, Adam P-M, Vial A, Royer P (2005). Phys Rev B.

[17] Billot L, Lamy de la Chapelle M, Grimault A-S, Vial A, Barchiesi D, Bijeon J-L, Adam P-M, Royer P (2006). Chem Phys Lett.

[18] D’Andrea C, Bochterle J, Toma A, Huck C, Neubrech F, Messina E, Fazio B, Maragò O M, Di Fabrizio E, Lamy de La Chapelle M (2013). ACS Nano.

[19] Cottat M, D’Andrea C, Yasukuni R, Malashikhina N, Grinyte R, Lidgi-Guigui N, Fazio B, Sutton A, Oudar O, Charnaux N (2015). J Phys Chem C.

[20] Tam F, Goodrich G P, Johnson B R, Halas N J (2007). Nano Lett.

[21] Bakker R M, Yuan H-K, Liu Z, Drachev V P, Kildishev A V, Shalaev V M, Pedersen R H, Gresillon S, Boltasseva A (2008). Appl Phys Lett.

[22] Fort E, Grésillon S (2008). J Phys D.

[23] Wang H, Kundu J, Halas N J (2007). Angew Chem, Int Ed.

[24] Neubrech F, Pucci A, Cornelius T W, Karim S, García-Etxarri A, Aizpurua J (2008). Phys Rev Lett.

[25] Neubrech F, Weber D, Enders D, Nagao T, Pucci A (2010). J Phys Chem C.

[26] Adato R, Yanik A A, Amsden J J, Kaplan D L, Omenetto F G, Hong M K, Erramilli S, Altug H (2009). Proc Natl Acad Sci U S A.

[27] Toma A, Tuccio S, Prato M, De Donato F, Perucchi A, Di Pietro P, Marras S, Liberale C, Zaccaria R P, De Angelis F (2015). Nano Lett.

[28] Osawa M, Ataka K, Yoshii K, Nishikawa Y (1993). Appl Spectrosc.

[29] Pucci A, Neubrech F, Weber D, Hong S, Toury T, Lamy de la Chapelle M (2010). Phys Status Solidi B.

[30] Aouani H, Šipová H, Rahmani M, Navarro-Cia M, Hegnerová K, Homola J, Hong M, Maier S A (2013). ACS Nano.

[31] Hillenbrand R, Taubner T, Keilmann F (2002). Nature.

[32] Marty R, Mlayah A, Arbouet A, Girard C, Tripathy S (2013). Opt Express.

[33] Cacciato G, Bayle M, Pugliara A, Bonafos C, Zimbone M, Privitera V, Grimaldi M G, Carles R (2015). Nanoscale.

[34] Milekhin A G, Yeryukov N A, Sveshnikova L L, Duda T A, Rodyakina E E, Gridchin V A, Sheremet E S, Zahn D R T (2015). Beilstein J Nanotechnol.

[35] Novotny L (2007). Phys Rev Lett.

[36] Bryant G W, Garcıa de Abajo F J, Aizpurua J (2008). Nano Lett.

[37] Palik E D (1998). Handbook of Optical Constants of Solids.

[38] (2016). High Frequency Structure Simulator. ANSYS, Inc..

[39] Ordal M A, Long L L, Bell R J, Bell S E, Bell R R, Alexander R W, Ward C A (1983). Appl Opt.

[40] Mooradian A, Wright G B (1966). Phys Rev Lett.

[41] Li Y B, Ferguson I T, Stradling R A, Zallen R (1992). Semicond Sci Technol.

